# Pulmonary Embolism as a Rare Complication of Clomiphene Therapy: A Case Report and Literature Review

**DOI:** 10.1155/2021/9987830

**Published:** 2021-09-07

**Authors:** Vinod Solipuram, Kishor Pokharel, Temple Ihedinmah

**Affiliations:** ^1^Department of Internal Medicine, Saint Agnes Hospital, Baltimore, MD 21229, USA; ^2^Saint George's University, Great River, NY 11739, USA

## Abstract

**Background:**

Pulmonary embolism (PE) is a medical emergency. Certain medications like oral contraceptives and hormone replacement therapies have been associated with PE. Clomiphene citrate is a selective estrogen receptor modulator, approved for treating infertility in females, but is being used as an off-label therapy in treating male hypogonadism. Very rarely, clomiphene can cause pulmonary embolism. *Case Report*. A 56-year-old gentleman presented with acute onset shortness of breath and chest discomfort. Upon further workup, he was found to have large volume pulmonary embolism. He was prescribed clomiphene citrate (CC) 2 years ago for hypogonadism. He was started on anticoagulation with improvement in his symptoms, and clomiphene was discontinued.

**Conclusion:**

Pulmonary embolism is a rare but potential complication of clomiphene therapy. In male patients with suspected hypogonadism, the risk of serious thromboembolic complications should be discussed before prescribing CC. Patients on CC have to be carefully monitored for serious side effects.

## 1. Introduction

Venous thromboembolism (VTE) refers to blood clots in the veins and comprises deep vein thrombosis (DVT) and pulmonary embolism (PE). PE refers to the blockage of the pulmonary arteries or its branches. Mostly, the blockage is by a thrombus, but obstruction by fat, air, and amniotic fluid emboli can also occur [1]. Very commonly, PE is a complication from DVT of the lower extremities. Certain conditions such as prolonged immobilization, cancer, obesity, and pregnancy are associated with increased risk of VTE [2]. In the general population, the incidence of PE is estimated to be around 60 to 70 per 100,000 [3]. A study estimated mortality rate upto 30% in untreated PE compared to 10% in treated PE [4]. The severity of PE is categorized as low, intermediate, and high risk based on hemodynamic stability and right ventricular dysfunction. Some medications like oral contraceptives, hormone replacement therapies, and certain chemotherapy regimens can increase the risk of VTE [2]. Clomiphene citrate (CC) is an FDA-approved medication used for treating infertility and hypogonadism in women. However, FDA has not approved its use in men with infertility or hypogonadism. But its use as off-label in male patients with hypogonadism has increased. The role of clomiphene in treating infertility among male patients is controversial. There have been few cases reported in the literature relating increased risk of VTE in association with CC. This includes reported cases of PE [5, 6], central retinal vein occlusion [7–9], and rarely intracranial (IC) thrombosis [10]. So far, only two case reports have been described attributing CC to IC thrombosis [10, 11] even without other major risk factors and may have increased risk if they have other contributing factors. The two reported cases are seen in young males within few weeks of using off-label clomiphene for infertility/hypogonadism.

Here, we present an uncommon case of pulmonary embolism in a middle-aged man on CC.

## 2. Case Presentation

A 56-year-old man presented to the emergency room with progressively worsening shortness of breath and chest discomfort of one week duration. He had associated nonproductive cough for one week but denied any calf pain, leg swelling, hemoptysis, recent travel, recent surgeries, fever, or chills. His past medical history was notable for hypertension that was well controlled on lisinopril. He was also prescribed with 50 mg of clomiphene citrate 2 years ago due to low testosterone. He did not take any other medications and denied any smoking history. He denied any family history of blood clots or bleeding disorders.

On physical examination, his temperature was 97.8 F, respiratory rate was 26 breaths/min, blood pressure was 109/58 mm Hg, heart rate was 134 beats/min, and SpO_2_ was 96% on 6 liters nasal cannula. He had normal heart sounds without any murmurs and rubs and had clear lungs. He had trace pedal edema bilaterally without any calf tenderness. Laboratory results include leukocytes of 15.4 × 10^9^/L, hemoglobin of 15.8 g/dL, platelets of 207 × 10^9^/L, creatinine of 2.2 mg/dL, and troponin I <0.05 ng/ml initially, but later plateaued at 0.10 ng/ml.

**Figure 1:**
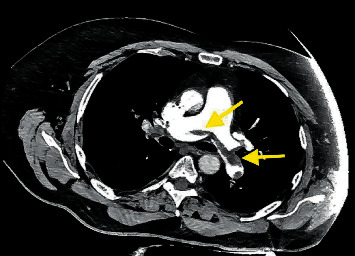
Computed tomography angiography (CTA) of the chest showing bilateral pulmonary embolism (yellow arrows).

Electrocardiogram on admission showed sinus tachycardia and incomplete right bundle branch block with S1Q3T3 pattern. Computed tomography angiography (CTA) of chest revealed acute large volume pulmonary embolism in bilateral main pulmonary arteries, lobar, and segmental branches of all lobes with mild right heart strain (as shown in Figure 1). Venous Doppler showed acute deep vein thrombosis in the right lower extremity involving the popliteal and posterior tibial veins. Echocardiogram was done which revealed a systolic ejection fraction of 55–65% with mildly decreased right ventricular systolic function.

The patient was started on a high flow nasal cannula initially for respiratory distress and then eventually was weaned to room air. He was started on heparin drip for anticoagulation that was later switched to rivaroxaban. Since clomiphene citrate (CC) was thought to be a contributing factor for pulmonary embolism, it was stopped, and he was referred to follow-up with a hematologist to evaluate for prothrombotic risk. His further workup for hypercoagulable disorders including anticardiolipin antibodies, factor V Leiden mutation and prothrombin gene mutation has been negative. He was also recommended to have age-appropriate screening for malignancy.

## 3. Discussion

The incidence of clomiphene-associated PE is extremely rare and is about <1% [12]. With the widespread use of clomiphene in recent years, the incidence of VTE (venous thromboembolism) is being increasingly recognized. Two case reports from 1986 and 2019 also published a similar event of pulmonary embolism in a male patient on clomiphene [5, 6].

Clomiphene citrate is a selective estrogen modulator that enhances the secretion of gonadotropin hormones by inhibiting the negative feedback from estradiol [8]. It has been used as the first-line treatment for induction of ovulation in women with anovulatory infertility. Increased gonadotropins can lead to increased androgens in men which in turn can improve hypogonadism. Depending upon the tissue location and levels of endogenous estrogen, clomiphene exhibits both agonist and antagonist activity. Due to its selective estrogen agonist activity, there is an increased risk of venous thromboembolism. Estrogen or its related compounds are known to increase the gene transcription of coagulation factors II, VII, VIII, X, XII, and fibrinogen which leads to increased coagulability [13]. In patients with underlying prothrombotic disorders like factor V Leiden mutation and malignancy, clomiphene can be a potential trigger for thrombotic events. In a systematic review done by Viola et al. [14], clomiphene has also been associated with increased visual disturbances and central retinal venous occlusion (CRVO). Deadly complications like cerebral venous thrombosis have also been reported with the use of clomiphene. Prior case reports on clomiphene-associated VTE are summarized in Table 1 [6, 7, 11–14].

There is no specific correlation between the timing of thrombotic events and initiation of clomiphene therapy. Cases of VTE have been reported as early as 3 weeks with initiation of the drug, but cases of central retinal venous occlusion (CRVO) and PE after several months have also been reported [8, 9].

Clomiphene use in the male population has conflicting results. Although some studies found clomiphene to be ineffective in treating idiopathic hypogonadism, reports where clomiphene successfully improved the sperm count and density also exist [15]. CC may not be as effective as testosterone replacement therapy in treating hypogonadism [16]. The first-line therapy in symptomatic male hypogonadism is testosterone therapy. Other side effects of clomiphene include nausea, vomiting, and visual disturbances. In female patients, ovarian enlargement and hyperstimulation syndrome have been reported. Rarely, pancreatitis, hypertriglyceridemia, and myocardial infarction were also noticed [14]. Prolonged use of clomiphene can also lead to an increased risk of ovarian cancer in females [17].

Treatment of clomiphene-associated PE includes stopping the drug and initiation of anticoagulation. For patients with hemodynamic instability and massive PE, thrombolysis should be considered. No data has been published to compare the efficacy and safety of various anticoagulation therapies with clomiphene-associated PE. Treatment with anticoagulation should be continued for at least 3–6 months, and repeat imaging or D-dimer is recommended in 3–6 months to assess for the resolution of clot [18]. Extensive workup for underlying prothrombotic disorders is also recommended, as these patients are particularly more susceptible to have thromboembolic events. All eligible patients should receive age-appropriate screening to rule out underlying malignancy. As the risk of VTE increases with age, CC must be used cautiously in patients more than 50 years [19].

## 4. Conclusion

Pulmonary embolism is a potential complication of clomiphene therapy. A careful discussion of such risk factors with the patients will play a crucial role in avoiding serious complications. More definitive prospective studies are required to assess the safety and efficacy of clomiphene use in men. Patients should be advised to improve physical activity while on clomiphene to prevent venous thromboembolism. Further workup to rule out prothrombotic disorders should be considered in all these patients.

## Figures and Tables

**Table 1:** Previously reported case reports of venous thrombosis associated with clomiphene.

Author name	Age/sex	Type of venous thrombosis	Duration of clomiphene use
Chamberlain and Cumming [5]	35 years/male	Pulmonary embolism	6 months
Lee [8]	36 years/female	Central retinal venous occlusion	8 months
Politou [9]	35 years/male	Central retinal venous occlusion	8 months
Viola [7]	38 years/female	Central retinal venous occlusion	1 month
Zahid [10]	Mid 30 s/male	Intracranial venous sinus thrombosis	3 weeks
Jeffrey Roan [6]	35 years/male	Pulmonary embolism	20 days

## Data Availability

Patient specific data could not be released as they are protected health information and in agreement with HIPAA regulations.

## Abbreviations

CC: Clomiphene citrate
CRVO: Central retinal vein occlusion
CTA: Computed tomography angiography
DVT: Deep vein thrombosis
F: Fahrenheit
FDA: Food and Drug Administration
g/dL: Gram per deciliter
L: Liter
mmHg: Millimeters of mercury
mg/dL: Milligram per deciliter
ng/ml: Nanogram per milliliter
PE: Pulmonary embolism
SpO2: Pulse oximetry
VTE: Venous thromboembolism.
